# Supplementary material for: HIVprotI: an integrated web based platform for prediction and design of HIV proteins inhibitors
Source: J Cheminform. 2018 Mar 9;10:12. doi: 10.1186/s13321-018-0266-y (PMC5845081; doi:10.1186/s13321-018-0266-y)
Supplement: Supplementary file 2 — Additional file 2. Source code of HIVProtI web server. [file 13321_2018_266_MOESM2_ESM.zip › HIVprotI_Source-code/hivprotI/weka-3-6-6/doc/weka/gui/ComponentHelper.html]

ComponentHelper


---


|  |  |  |
| --- | --- | --- |
| **Overview** \| **Package** \| **Class** \| **Tree** \| **Deprecated** \| **Index** \| **Help** \| **Weka's home** | |  |
| **PREV CLASS**   **NEXT CLASS** | **FRAMES**    **NO FRAMES**     **All Classes** |  |
| SUMMARY: NESTED \| FIELD \| CONSTR \| METHOD | DETAIL: FIELD \| CONSTR \| METHOD |  |


---


## weka.gui Class ComponentHelper

```
java.lang.Object
  weka.gui.ComponentHelper
```

---

``` public class ComponentHelper extends java.lang.Object ```

A helper class for some common tasks with Dialogs, Icons, etc.

**Version:**
:   $Revision: 7059 $

**Author:**
:   FracPete (fracpete at waikato dot ac dot nz)

---

| **Field Summary** | |
| --- | --- |
| `static java.lang.String[]` | `IMAGES`             the default directories for images |


**Constructor Summary**

`ComponentHelper()`


| **Method Summary** | |
| --- | --- |
| `static java.awt.Image` | `getImage(java.lang.String filename)`             returns the Image for a given filename, NULL if not successful |
| `static java.awt.Image` | `getImage(java.lang.String dir, java.lang.String filename)`             returns the Image for a given directory and filename, NULL if not successful |
| `static javax.swing.ImageIcon` | `getImageIcon(java.lang.String filename)`             returns the ImageIcon for a given filename, NULL if not successful |
| `static javax.swing.ImageIcon` | `getImageIcon(java.lang.String dir, java.lang.String filename)`             returns the ImageIcon for a given filename and directory, NULL if not successful |
| `static java.lang.String` | `showInputBox(java.awt.Component parent, java.lang.String title, java.lang.String msg, java.lang.Object initialValue)`             pops up an input dialog |
| `static int` | `showMessageBox(java.awt.Component parent, java.lang.String title, java.lang.String msg, int buttons, int messageType)`             displays a message box with the given title, message, buttons and icon ant the dimension. |

**Methods inherited from class java.lang.Object**

`equals, getClass, hashCode, notify, notifyAll, toString, wait, wait, wait`

**Field Detail**

### IMAGES

```
public static final java.lang.String[] IMAGES
```

:   the default directories for images


**Constructor Detail**

### ComponentHelper

```
public ComponentHelper()
```


**Method Detail**

### getImageIcon

```
public static javax.swing.ImageIcon getImageIcon(java.lang.String dir,
                                                 java.lang.String filename)
```

:   returns the ImageIcon for a given filename and directory, NULL if not successful

    :   **Parameters:**: `dir` - the directory to look in for the file: `filename` - the file to retrieve **Returns:**: the imageicon if found, otherwise null

---


### getImageIcon

```
public static javax.swing.ImageIcon getImageIcon(java.lang.String filename)
```

:   returns the ImageIcon for a given filename, NULL if not successful

    :   **Parameters:**: `filename` - the file to retrieve **Returns:**: the imageicon if found, otherwise null

---


### getImage

```
public static java.awt.Image getImage(java.lang.String dir,
                                      java.lang.String filename)
```

:   returns the Image for a given directory and filename, NULL if not successful

    :   **Parameters:**: `dir` - the directory to look in for the file: `filename` - the file to retrieve **Returns:**: the image if found, otherwise null

---


### getImage

```
public static java.awt.Image getImage(java.lang.String filename)
```

:   returns the Image for a given filename, NULL if not successful

    :   **Parameters:**: `filename` - the file to retrieve **Returns:**: the image if found, otherwise null

---


### showMessageBox

```
public static int showMessageBox(java.awt.Component parent,
                                 java.lang.String title,
                                 java.lang.String msg,
                                 int buttons,
                                 int messageType)
```

:   displays a message box with the given title, message, buttons and icon
    ant the dimension.
    it returns the pressed button.

    :   **Parameters:**: `parent` - the parent component: `title` - the title of the message box: `msg` - the text to display: `buttons` - the captions of the buttons to display: `messageType` - the type of message like defined in `JOptionPane` (the icon is determined on this basis) **Returns:**: the button that was pressed **See Also:**: `JOptionPane`

---


### showInputBox

```
public static java.lang.String showInputBox(java.awt.Component parent,
                                            java.lang.String title,
                                            java.lang.String msg,
                                            java.lang.Object initialValue)
```

:   pops up an input dialog

    :   **Parameters:**: `parent` - the parent of this dialog, can be `null`: `title` - the title to display, can be `null`: `msg` - the message to display: `initialValue` - the initial value to display as input **Returns:**: the entered value, or if cancelled `null`


---


|  |  |  |
| --- | --- | --- |
| **Overview** \| **Package** \| **Class** \| **Tree** \| **Deprecated** \| **Index** \| **Help** \| **Weka's home** | |  |
| **PREV CLASS**   **NEXT CLASS** | **FRAMES**    **NO FRAMES**     **All Classes** |  |
| SUMMARY: NESTED \| FIELD \| CONSTR \| METHOD | DETAIL: FIELD \| CONSTR \| METHOD |  |


---
